# A modified Bilirubin-induced neurologic dysfunction (BIND-M) algorithm is useful in evaluating severity of jaundice in a resource-limited setting

**DOI:** 10.1186/s12887-015-0355-2

**Published:** 2015-04-01

**Authors:** Paula G Radmacher, Frank D Groves, Joshua A Owa, Gabriel E Ofovwe, Emmanuel A Amuabunos, Bolajoko O Olusanya, Tina M Slusher

**Affiliations:** Department of Pediatrics, University of Louisville School of Medicine, Louisville, KY USA; Department of Epidemiology and Population Health, University of Louisville School of Public Health and Information Sciences, Louisville, KY USA; Department of Paediatrics and Child Health, Obafemi Awolowo University, Ile-Ife, 220005 Nigeria; Department of Child Health, University of Benin, Benin City, Nigeria; Neonatal Unit, University of Benin Teaching Hospital, Benin City, Nigeria; Centre for Healthy Start Initiative, Ikoyi, Lagos Nigeria; Center for Global Pediatrics, University of Minnesota, Minneapolis, MN USA; Hennepin County Medical Center, Minneapolis, MN USA

**Keywords:** Severe neonatal jaundice, Acute bilirubin encephalopathy, Evaluation of BIND score

## Abstract

**Background:**

Severe neonatal jaundice with associated acute bilirubin encephalopathy occurs frequently in low- and middle-income countries, where advanced diagnostic technology is in short supply. In an effort to facilitate the physical diagnosis of acute bilirubin encephalopathy, we pilot-tested a modified bilirubin induced neurologic dysfunction scoring algorithm in a group of pediatric trainees (residents) and their mentors (consultants) in a resource-constrained setting.

**Methods:**

Jaundiced Nigerian infants were examined by consultant and resident pediatricians. The modified bilirubin induced neurologic dysfunction score assigned by residents was compared with the clinical diagnosis of acute bilirubin encephalopathy by expert consultants. Demographic information was obtained. Known risk factors were also evaluated among infants with and without acute bilirubin encephalopathy in addition to exploratory analyses. Data were analyzed by Statistical Analysis System; statistical significance was set at p < 0.05.

**Results:**

Three hundred and thirty three paired modified bilirubin induced neurologic dysfunction scores (333) were analyzed and showed excellent agreement (weighted Kappa coefficient 0.7969) between residents and consultants. A modified bilirubin induced neurologic dysfunction score greater than or equal to 3 was highly predictive of a clinical diagnosis of acute bilirubin encephalopathy, with sensitivity of 90.7%, specificity of 97.7%, positive predictive value of 88.9%, and negative predictive value of 98.2%. Exposure to mentholated products was strongly associated with increased risk of acute bilirubin encephalopathy among those with known glucose-6-phosphate dehydrogenase deficiency (odds ratio = 73.94; 95% confidence interval = 5.425-infinity) as well as among those whose G6PD phenotype was unknown (odds ratio = 25.88; 95% confidence interval = 2.845-235.4).

**Conclusions:**

The modified bilirubin induced neurologic dysfunction score for neonatal jaundice can be assigned reliably by both residents and experienced pediatricians in resource-limited settings as reflected in the algorithm’s sensitivity and specificity. It may be useful for predicting the development and severity of acute bilirubin encephalopathy in neonates.

**Electronic supplementary material:**

The online version of this article (doi:10.1186/s12887-015-0355-2) contains supplementary material, which is available to authorized users.

## Background

Worldwide, neonatal jaundice (NNJ) is one of the most common conditions requiring in-patient treatment in the early postnatal period [1-3]. Successful early detection and postnatal treatment procedures before and after hospital discharge have made the potentially lethal neurotoxic effects of severe neonatal jaundice a rare occurrence in the industrialized world [4,5]. For instance, it is unusual to treat neonatal jaundice beyond phototherapy except in a few extreme cases when exchange blood transfusion (EBT) may be required to quickly reduce the circulating unconjugated bilirubin concentrations and prevent the movement of free bilirubin into the brain tissues.

In resource-limited settings however, neonatal jaundice continues to be a major cause of neonatal mortality as well as morbidity, characterized by acute bilirubin encephalopathy (ABE), and later, kernicterus in the form of auditory neuropathy and chorioathetoid cerebral palsy, and mortality [6]. In Nigeria where the burden of neonatal jaundice is exceptionally high, many infants are born at home or church clinics, and may die before ever seeing a physician or other health care workers [7,8]. Neonatal jaundice is often not easily appreciated by mothers and caregivers in the home setting until it becomes severe enough to deeply discolor the sclera and the skin, at which point neurological damage may have already occurred. Additionally, a considerable number of infants arrive at the hospital or health care facility only after irreversible bilirubin encephalopathy has occurred [8]. In a recently published survey, only 51.5% of community health workers in southwest Nigeria gave a correct definition of neonatal jaundice, and only 45.5% correctly identified danger signs [9]. Even trained medical professionals may miss the clinical signs and features of ABE, especially in the early stages, so that appropriate intervention for infants with reversible brain injury may be delayed [10,11]. Thus there is a need for a tool that clinicians can use to recognize the danger signs of ABE early enough when appropriate intervention is most likely to lead to limiting or reversing adverse neurodevelopmental outcomes.

In resource-limited settings where the diagnosis of ABE is commonly made without magnetic resonance imaging (MRI) or auditory brainstem response (ABR) confirmation, experienced pediatricians are adept at recognizing this constellation of signs. However, attempts to quantify the prevalence of ABE and its severity across communities and among physicians with different levels of expertise were historically limited by the lack of a uniform diagnostic algorithm. In 1999, the bilirubin-induced neurologic dysfunction (BIND) scoring algorithm was developed, assigning 1, 2 or 3 points to indicate mild, moderate, or severe abnormalities in an infant’s mental status, muscle tone, or cry (Additional file 1) [12]. Higher scores indicate worsening signs of acute neurotoxicity associated with excessive hyperbilirubinemia, providing a common descriptive framework for clinicians and researchers to estimate the severity of neonatal jaundice. The simplicity of the BIND scoring system, similar to the Apgar score, makes it an attractive tool for clinical diagnosis of ABE in resource limited locations that lack ready access to MRI) and ABR testing [13]. However, the practicality and acceptance of this scoring algorithm in such settings has not been reported. This study was therefore designed to evaluate the reliability and reproducibility of a modified BIND (BIND-M) scoring system within the scope of the routine newborn examination in Nigeria when used by both trainees and consultant pediatricians. Additionally, BIND-M scores were analyzed for utility in a subset of infants with recorded clinical signs and symptoms of varying degrees of jaundice.

## Methods

### Human subjects

This study was approved by the Institutional Review Boards (IRBs) at the University of Louisville USA, the University of Minnesota USA, the Obafemi Awolowo University Teaching Hospitals Complex, Nigeria, the Ladoke Akintola University of Technology Teaching Hospital, Nigeria and the University of Benin Teaching Hospital, Nigeria prior to its initiation.

### Modified BIND score

With the permission of Drs. Lois Johnson and Vinod Bhutani, the authors of the original BIND scoring algorithm (Additional file 1), the investigators developed a modified BIND scoring algorithm (BIND-M), which incorporates abnormality of the upward gaze, another classic sign of bilirubin encephalopathy, with additional qualifiers (Additional file 2). The goal was to develop a more comprehensive score which would better discriminate degrees of BIND severity. In the new scoring system, the maximum total score for BIND-M is 12. A score of 1-4 was predicted to be indicative of mild ABE, which is generally considered to be reversible if treated promptly and aggressively. An intermediate score (5-6) was predicted to be indicative of moderate ABE, which might be reversible with urgent and prompt bilirubin reduction. Even higher scores (7 and above) would likely indicate severe/very severe ABE, probably representing irreversible brain damage in most infants.

### Data collection

One of the authors (TMS) provided initial training in the BIND-M scoring system to the consultant pediatricians at the sites, who in turn trained the resident physicians. The BIND-M was then incorporated into the routine physical examination of jaundiced infants in the hospital nurseries by both resident and consultant pediatricians, who independently computed a BIND-M score for each of 333 infants. Regardless of the score, physicians also were asked to determine if they believed an infant had the signs and symptoms of ABE, based on the total clinical picture.

The BIND-M score and clinical determination of ABE by the consultant pediatricians were considered to be the reference points for this study. The total BIND-M scores, as assessed by residents, were cross-tabulated against the total BIND-M scores as assessed by consultants. Additionally, resident and consultant findings for each major sub-score of the BIND-M (alterations in muscle tone, mental status, cry, and gaze) were separately cross-tabulated. For each cross-tabulation, inter-rater agreement was assessed using the weighted Kappa statistic, with 95% confidence intervals (CI), as computed using SAS PROC FREQ.

Descriptive and exploratory analyses were conducted using additional clinical variables where available, as well as known risk factors for neonatal jaundice (sepsis, feto-maternal blood groups incompatibility, G6PD deficiency, menthol exposure). Means of continuous variables were compared between infants with and without ABE using SAS PROC MEANS and PROC T-TEST. Statistical significance was set at p < 0.05. Odds ratios (ORs) for dichotomous and categorical variables, along with their 95% confidence intervals were calculated using unconditional logistic regression (SAS PROC LOGISTIC). Discordance at the A, B, and Rh loci were evaluated simultaneously in a single mode. Likewise, menthol exposure was evaluated in the context of G6PD deficiency, such that infants who were neither enzyme deficient nor exposed to menthol were the “grand” referent group to which all others were compared.

## Results

### Inter-rater reliability of the BIND-M score: residents versus consultants

Inter-rater reliability of BIND-M between pediatric residents and consultant pediatricians was determined for 333 records (Table 1). The total BIND-M scores showed excellent agreement between residents and consultants, as confirmed by the weighted Kappa coefficient of 0.7969 (95% CI, 0.7426, 0.8512). The sub-scores for mental status (Table 2a), muscle tone (Table 2b), and cry (Table 2c), likewise showed a high level of agreement, with weighted Kappa coefficients of 0.7692 (95% CI: 0.6977, 0.8407), 0.8430 (95% CI: 0.7809, 0.9051), and 0.8139 (95% CI: 0.7321, 0.8957), respectively. Only the simple Kappa coefficient of 0.5802 (95% CI: 0.4055, 0.7549) could be computed for the gaze sub-score (Table 2d), since it can take on only two values: zero and three.

**Table 1:** **BIND-M scores: resident versus consultant pediatricians**

**BIND-M score**		**Consultants**													
		**0**	**1**	**2**	**3**	**4**	**5**	**6**	**7**	**8**	**9**	**10**	**11**	**12**	**Total**
Residents	0	236	11	4	1	0	0	0	1	0	1	0	0	0	254
	1	8	7	1	0	0	0	0	0	0	0	0	0	0	16
	2	3	2	1	1	0	1	0	0	0	0	0	0	0	8
	3	0	1	1	1	0	0	1	0	0	1	0	0	0	5
	4	0	0	0	1	0	1	0	1	0	1	0	0	0	4
	5	1	0	0	0	0	1	0	1	0	1	1	0	0	5
	6	0	0	1	0	1	1	2	0	0	0	0	1	0	6
	7	0	0	0	0	1	1	0	2	0	0	0	0	0	4
	8	0	2	0	0	0	1	0	1	3	1	2	2	0	12
	9	0	0	0	0	0	0	0	0	0	2	1	0	0	3
	10	0	0	0	0	0	0	0	0	1	2	2	0	0	5
	11	0	0	0	0	0	0	0	0	3	0	0	5	0	8
	12	0	0	0	0	0	0	0	0	0	0	0	1	2	3
	Total	248	23	8	4	2	6	3	6	7	9	6	9	2	333

Weighted Kappa = 0.7969 (95% CI: 0.7426, 0.8512).

**Table 2:** **Sub-score correlations of BIND-M scores: residents versus consultants**

**a: Mental status sub-scores**			**Consultants**				
**Weighted Kappa = 0.7692 (95% CI: 0.6977, 0.8407)**			**0**	**1**	**2**	**3**	**Total**
Residents	Normal mental status.	0	246	11	3	1	261
	Sleepy, Difficult to Awaken for Feeding	1	12	10	2	0	24
	Very sleepy; Alternatively very irritable	2	3	1	27	1	32
	Semi-Coma; Apnea; Convulsions	3	0	2	5	9	16
	Total		261	24	37	11	333
**b: Muscle tone sub-scores**			**Consultants**				
**Weighted Kappa = 0.8430 (95% CI: 0.7809, 0.9051)**			**0**	**1**	**2**	**3**	**Total**
Residents	Normal muscle tone	0	259	7	3	1	270
	Persistent mild hypotonia	1	2	7	0	0	9
	Moderate hypotonia; moderate hypertonia; increasing arching of neck and trunk on stimulation without spasms of arms & legs and without trismus	2	3	3	22	4	32
	Persistent retrocollis; opisthotonus; crossing or scissoring of arms or legs but without spasms of arms & legs and without trismus	3	1	0	3	18	22
	Total		265	17	28	23	333
**c: Altered cry sub-scores**			**Consultants**				
**Weighted Kappa = 0.8139 (95% CI: 0.7321, 0.8957)**			**0**	**1**	**2**	**3**	**Total**
Residents	Normal cry	0	279	3	1	3	286
	High-pitched cry	1	4	2	1	3	10
	Shrill cry	2	1	0	6	0	7
	Inconsolable crying, or cry weak or absent in child with previous history of high pitched or shrill cry	3	2	1	3	24	30
	Total		286	6	11	30	333
**d: Altered gaze sub-scores**			**Consultants**				
**Simple Kappa = 0.5802 (95% CI: 0.4055, 0.7549)**			**0**	**3**			**Total**
Residents	Normal Gaze	0	301	12			313
	Sun-setting; paralysis of upward gaze	3	6	14			20
	Total		307	26			333

The consultant deemed 53 (15.9%) of the 333 infants to have ABE. Total BIND-M scores were evaluated for clinical utility in diagnosing ABE (Table 3). An overall score greater than or equal to 3 was highly predictive of a clinical diagnosis of ABE, with sensitivity of 90.7%, specificity of 97.7%, positive predictive value of 88.9%, and negative predictive value of 98.2%. Fifty (94.3%) of the 53 infants with ABE had scores of “2” (“moderate”) or “3” (“severe”) on at least one subscale (mental status, muscle tone, cry, or eye findings). However, residents had 9 false positives versus 8 for consultants, based on this single criterion. The most consistently detected sign was alteration in muscle tone. Residents scored 48 of 53 (90.6%) infants with ABE in the “moderate-to-severe” range; the consultants similarly scored 84.9% of these infants. Alteration in mental status was a less consistent finding for ABE; residents noted “moderate-to-severe” alterations in mental status for 42 out of the 53 (79.2%) infants with ABE versus 44 (83%) noted by the consultants. Alterations in cry were similarly noted by residents and consultants (44 and 43 of 53, respectively). Consultants recognized paralysis of upward gaze in 26 (49%) of the 53 infants with encephalopathy, versus only 19 (35.8%) that were detected by residents. All infants with eye findings had a diagnosis of ABE. However, when eye finding points were subtracted from the BIND-M score, all infants with ABE still had a BIND score of 3 and above.

**Table 3:** **BIND-M score versus consultant diagnosis of acute bilirubin encephalopathy (ABE)**

**Consultant score vs. consultant diagnosis**		**Final diagnosis**				**Test performance**			
		**ABE**		**No ABE**		**Sensitivity %**	**Specificity %**	**Positive predictive value %**	**Negative predictive value %**
		**TP**	**FN**	**FP**	**TN**				
**BIND score cut off**	**≥0**	**53**	**0**	**280**	**0**	**100.0**	**0.0**	**15.9**	**N/A**
	**≥1**	**52**	**1**	**33**	**247**	**98.1**	**88.2**	**61.2**	**99.6**
	**≥2**	**50**	**3**	**12**	**268**	**94.3**	**95.7**	**80.6**	**98.9**
	**≥3**	**48**	**5**	**6**	**274**	**90.7**	**97.9**	**88.9**	**98.2**
	**≥4**	**45**	**8**	**5**	**275**	**84.9**	**98.2**	**90.0**	**97.2**
	**≥5**	**44**	**9**	**4**	**276**	**83.0**	**98.6**	**91.7**	**96.8**
	**≥6**	**40**	**13**	**2**	**278**	**75.5**	**99.3**	**95.2**	**95.5**
	**≥7**	**37**	**16**	**2**	**278**	**69.8**	**99.3**	**94.6**	**94.9**
	**≥8**	**32**	**21**	**1**	**279**	**60.4**	**99.6**	**96.7**	**93.0**
	**≥9**	**26**	**27**	**0**	**280**	**49.6**	**100.0**	**100.0**	**91.5**
	**≥10**	**17**	**36**	**0**	**280**	**32.1**	**100.0**	**100.0**	**88.6**
	**≥11**	**11**	**42**	**0**	**280**	**20.8**	**100.0**	**100.0**	**87.0**
	**≥12**	**2**	**51**	**0**	**280**	**3.8**	**100.0**	**100.0**	**84.6**

### Risk factors for neonatal jaundice

A total of 198 records met criteria for additional exploratory analyses. Pediatric consultants made a clinical diagnosis of ABE in 28 (14.1%) of these 198 infants. Peak serum bilirubin concentrations were almost twice as high in infants with ABE compared to those without ABE, and peak bilirubin > 15 mg/dL was associated with a tenfold OR for ABE (Table 4). Sixty-nine percent of infants were term (≥38 weeks), with a mean estimated gestational age of 37.7 (2.7) weeks, and 82.3% were born either at a clinic or in a hospital. The mean age at the time of BIND-M examination was 124 (74) hours; the mean infant weight at the time of examination was 2751 (725) grams. In infants with a clinical diagnosis of ABE, the mean postnatal age at examination was higher than in infants without ABE but not statistically significant (p = 0.053). Additionally, gestational age at birth, birth weight, weight at examination and the proportions of infants with ABO and Rh blood incompatibilities did not differ significantly between the groups.

**Table 4:** **Risk factors for acute Bilirubin encephalopathy among Jaundiced Nigerian neonates**

**Peak serum bilirubin**		**Cases**	**Controls**	**Number (%)**	**Odds ratio [95% CI]**
<=15.00		3	93	96 (28.8%)	1.000 [Referent]
Unknown		25	110	135 (40.5%)	7.044 [2.061,24.07]
>15.00		77	25	102 (30.6%)	10.06 [2.927,34.60]
Total		105	228	333 (100.0%)	
Means		30.71	15.65		P < 0.001
**Gender**		**Cases**	**Controls**	**Number (%)**	**Odds Ratio (95% CI)**
Male		17	103	120 (61.2%)	1.089 (0.470,2.523)
Female		10	66	76 (38.8%)	1.000 (REFERENT)
**Sepsis**		**Cases**	**Controls**	**Number (%)**	**Odds Ratio (95% CI)**
No		4	67	71 (66.4%)	1.000 (REFERENT)
Yes		12	24	36 (33.6%)	8.375 (2.463, 28.48)
**Menthol**	**G6PD**	**Cases**	**Controls**	**Number (%)**	**Odds Ratio (95% CI)**
Unexposed	Normal	1	37	38 (19.2%)	1.000 (REFERENT)
Unexposed	Unknown	10	63	73 (36.9%)	5.868 (0.722,47.66)
Unexposed	Deficient	3	17	20 (10.1%)	6.524 (0.632,67.34)
Unknown	Normal	1	10	11 (5.6%)	3.697 (0.212,64.42)
Unknown	Unknown	1	25	26 (13.1%)	7.120 (0.936,54.18)
Unknown	Deficient	1	6	7 (3.5%)	6.162 (0.338,112.2)
Exposed	Normal	0	0	0 (0.0%)	0.000 (incalculable)
Exposed	Unknown	7	10	17 (8.6%)	25.88 (2.845,235.8)
Exposed	Deficient	4	2	6 (3.0%)	73.94 (5.425,>999)

One hundred seven of the jaundiced infants were evaluated for sepsis. Among these, sepsis was diagnosed in 24 (26.4%) of 91 without ABE and in 12 (75.0%) of 16 with ABE (OR = 8.375; 95% CI = 2.463, 28.48). Finally, when ABE was cross-tabulated simultaneously with menthol exposure and G6PD deficiency (Table 4), it was clear that menthol exposure was strongly associated with ABE, but statistically significant only among those whose known G6PD phenotype was unknown (OR = 25.88; 95% CI = 2.845,235.8) or definitely deficient (OR = 73.94; 95% CI = 5.425, > 999).

## Discussion

Our results have shown that the physical diagnosis of ABE can be facilitated by a modified BIND-M score. The score showed excellent agreement between residents and consultants (weighted Kappa = 0.7969), and appeared to be accurate in predicting infants with ABE (sensitivity = 90.7%; positive predictive value = 88.9%). Thus, BIND-M may be an important yet simple tool to provide a uniform measure of the degree of ABE in clinically jaundiced infants in resource limited settings. Infants with BIND-M scoring less than 3 were extremely unlikely to be diagnosed with clinical ABE (specificity = 97.7%; negative predictive value = 98.2%). Although, a score of 5 had a higher positive predictive value of 91.7%, it is associated with an unacceptable level of false negatives (80% increase) with an 8% reduction in sensitivity. While we predicted that a score of 1-4 would be indicative of mild ABE and 5-6 would represent moderate ABE, a threshold as low as 3 is definitely more applicable in this population, possibly as a result of the multiple risk factors and a high prevalence of severe NNJ. It is noteworthy that inter-rater agreement for the upward gaze paralysis sub-score (simple Kappa = 0.5802) was lower than that for the altered mental status (weighted Kappa = 0.7692), altered muscle tone (weighted Kappa = 0.8430), and altered cry (weighted Kappa = 0.81399) sub-scores.

Exchange blood transfusion for severe NNJ entails a significant risk for morbidity. This procedure was quite common in the present study, where 24% of the infants at four major teaching hospitals in Nigeria had EBT. Even in the most experienced hands, this procedure is not without significant risk from the procedure itself as well as the risks for infections and non-infectious complications [14,15]. If effectively used in combination with other modalities including intensive phototherapy, BIND-M is capable of reducing the high rate of needless EBT in newborns without ABE as well as enhancing identification of newborns with mild to moderate ABE, considered to be reversible with appropriate and early interventions.

Glucose-6-phosphate dehydrogenase deficiency is known to be a significant risk factor for severe neonatal jaundice and is common in Nigeria. Testing for G6PD deficiency is not routinely available in health institutions and was only performed in a small subset of infants in the present study. Exposure to mentholated products is known to be a significant risk factor for severe jaundice and ABE in infants with G6PD deficiency [16,17], and yet was commonly used with the neonates in our study: 1 in 6 infants overall, and 4 in 10 infants with ABE. It is therefore imperative to continue public enlightenment campaigns, at least in Nigeria, to educate the populace, and pregnant women in particular, as to the danger of using these products in the newborn period [17].

Surprisingly, neither ABO incompatibility nor Rh disease was significantly different between the two groups of infants with and without ABE, suggesting that other factors such as dehydration, sepsis and harmful cultural practices may play a more significant role in determining the course and severity of newborn jaundice in this area. Concurrent sepsis has been alleged to be a significant risk factor for ABE among Nigerian neonates [18]. Sepsis usually coexists with exposure to exogenous agents that predispose the G6PD deficient infants to severe NNJ and ABE.

The primary limitation in the present study is the lack of ABR or MRI to validate the BIND-M score. However, since neither ABR nor MRI is the standard of care in these settings, physicians are quite familiar with the clinical diagnosis of ABE. A subsequent study to validate the BIND-M score with ABR is now ongoing in a children’s hospital in Lagos, Nigeria. Another significant limitation is the lack of the full medical history for these infants. However, since this was not the focus of this study it will be desirable for a future study.

## Conclusions

The present study has shown that the use of the BIND-M score among consultant pediatricians and their resident doctors in Nigeria is reliable for identifying infants with the clinical diagnosis of ABE with acceptable sensitivity and specificity. Validation and further modification of this scoring system could provide a much needed tool for use in population studies in Nigeria and elsewhere to estimate the actual magnitude of ABE-related morbidity and mortality. Such data would support the work of child health advocates to devise solutions to reduce or eliminate this preventable morbidity which has long-term, tragic consequences for the infants, their families and their communities.

## Additional files

Additional file 1:
**Original BIND score scheme.**
Additional file 2:
**Clinical format for prospective scoring of onset, severity and duration of ABE in clinically Jaundiced infants-modified BIND (BIND-M).**

## Abbreviations

ABE: Acute bilirubin encephalopathy
BIND-M: Modified bilirubin induced neurologic dysfunction
G6PD: Glucose-6-phosphate dehydrogenase
SAS: Statistical analysis system
NNJ: Neonatal jaundice
EBT: Exchange blood transfusion
MRI: Magnetic resonance imaging
ABR: Auditory brainstem response
OR: Odds ratio
CI: Confidence interval
