# Supplementary material for: A modified Bilirubin-induced neurologic dysfunction (BIND-M) algorithm is useful in evaluating severity of jaundice in a resource-limited setting
Source: BMC Pediatr. 2015 Apr 1;15:28. doi: 10.1186/s12887-015-0355-2 (PMC4389967; doi:10.1186/s12887-015-0355-2)
Supplement: Additional file 1: — Original BIND score scheme. [file 12887_2015_355_MOESM1_ESM.docx]

**Additional file 1**. Original BIND score scheme.

| **BIND SCORE** | **Stage 1A**  **Early**  **(1 point)** | **Stage 1B**  **Moderate**  **(2 points)** | **Stage II**  **Severe, Semi-Coma, Apnea, Convulsions**  **(3 points)** |
| --- | --- | --- | --- |
| **Mental Status**  **(circle one)** | Sleepy,  Difficult to Awaken for Feeding | Very sleepy  Alternatively very irritable | Semi-Coma  Apnea  Convulsions |
| **Muscle Tone**  **(circle one)** | Tone slightly decreased | Tone moderately increased or decreased depending on arousal state. Mild neck and back arching | Tone markedly increased or decreased. Opisthotonic posturing. “Back bends” “Bicycling” movements |
| **Cry**  **(Circle one)** | High pitched | Shrill, very high pitched | Piercing, shrill inconsolable |
| **Suck**  **(Circle one)** |  | Weak/Poor | Absent |
| ***Feeding**  **(Circle One)** | Decreased | Poor | Absent |
| ****Yellowness (at any time)**  **(Circle One)** | Eyes/Face | Trunk/Chest | Abdomen and Below  ** (Yellowness not scored but included in record) |
