# Supplementary material for: A modified Bilirubin-induced neurologic dysfunction (BIND-M) algorithm is useful in evaluating severity of jaundice in a resource-limited setting
Source: BMC Pediatr. 2015 Apr 1;15:28. doi: 10.1186/s12887-015-0355-2 (PMC4389967; doi:10.1186/s12887-015-0355-2)
Supplement: Additional file 2: — Clinical format for prospective scoring of onset, severity and duration of ABE in clinically Jaundiced infants-modified BIND (BIND-M). [file 12887_2015_355_MOESM2_ESM.docx]

**Additional file 2.** Clinical Format for Prospective Scoring of Onset, Severity and Duration of ABE in Clinically Jaundiced Infants-Modified BIND (BIND-M)

| **CLINICAL SIGN** | **SCORE** | **SEVERITY** | Date/Time |
| --- | --- | --- | --- |
| **MENTAL STATUS** | | |  |
| Normal | 0 | None |  |
| Sleepy but arousable  Decreased feeding | 1 | Mild |  |
| Lethargy  Poor suck and/or  Irritable/jittery with short-term strong suck | 2 | Moderate |  |
| Semi-coma  Apnea  Seizures  Coma | 3 | Severe |  |
| **MUSCLE TONE** | | | |
| Normal | 0 | None |  |
| Persistent mild hypotonia | 1 | Mild |  |
| Moderate hypotonia  Moderate hypertonia  Increasing arching of neck and trunk on stimulation without spasms of arms and legs and without trismus | 2 | Moderate |  |
| Persistent retrocolis  Opisthotonus  Crossing or scissoring of arms or legs but without spasms of arms and legs and without trismus | 3 | Severe |  |
| **CRY PATTERN** | | | |
| Normal | 0 | None |  |
| High pitched | 1 | Mild |  |
| Shrill | 2 | Moderate |  |
| Inconsolable crying or  Cry weak or absent in child with previous  history of high pitched or shrill cry | 3 | Severe |  |
| **OCCULOMOTOR OR EYE MOVEMENTS** | | | |
| Normal | 0 | None, Mild |  |
| Sun-setting  Paralysis of Upward Gaze | 3 | Severe |  |
| **Total ABE Score** |  |  | |
| MD signature: | | I believe this infant has clinical signs/symptoms of ABE despite BIND score. | |
